# Mucopolysaccharidosis IVA: Diagnosis, Treatment, and Management

**DOI:** 10.3390/ijms21041517

**Published:** 2020-02-23

**Authors:** Kazuki Sawamoto, José Víctor Álvarez González, Matthew Piechnik, Francisco J. Otero, Maria L. Couce, Yasuyuki Suzuki, Shunji Tomatsu

**Affiliations:** 1 Nemours/Alfred I. duPont Hospital for Children, Wilmington, DE 19803, USA; kz.sawamoto@gmail.com (K.S.); Josevictor.Alvarezgonzalez@nemours.org (J.V.Á.G.); Matt.Piechnik@nemours.org (M.P.); 2 University of Delaware, Newark, DE 19716, USA; 3 Department of Pharmacology, Pharmacy and Pharmaceutical Technology, School of Pharmacy, University of Santiago de Compostela, 15872 Santiago de Compostela, Spain; francisco.otero@usc.es; 4 Department of Forensic Sciences, Pathology, Gynecology and Obstetrics and Pediatrics Neonatology Service, Metabolic Unit, IDIS, CIBERER, MetabERN, University Clinic Hospital of Santiago de Compostela, 15706 Santiago de Compostela, Spain; Maria.Luz.Couce.Pico@sergas.es; 5 Department of Pediatrics, Graduate School of Medicine, Gifu University, Gifu 501-1193, Japan; ysuz@gifu-u.ac.jp; 6 Department of Pediatrics, Thomas Jefferson University, Philadelphia, PA 19107, USA

**Keywords:** MPS IVA, GALNS, keratan sulfate, skeletal dysplasia, LC-MS/MS, bone-targeting, tracheal reconstructive surgery

## Abstract

Mucopolysaccharidosis type IVA (MPS IVA, or Morquio syndrome type A) is an inherited metabolic lysosomal disease caused by the deficiency of the N-acetylglucosamine-6-sulfate sulfatase enzyme. The deficiency of this enzyme accumulates the specific glycosaminoglycans (GAG), keratan sulfate, and chondroitin-6-sulfate mainly in bone, cartilage, and its extracellular matrix. GAG accumulation in these lesions leads to unique skeletal dysplasia in MPS IVA patients. Clinical, radiographic, and biochemical tests are needed to complete the diagnosis of MPS IVA since some clinical characteristics in MPS IVA are overlapped with other disorders. Early and accurate diagnosis is vital to optimizing patient management, which provides a better quality of life and prolonged life-time in MPS IVA patients. Currently, enzyme replacement therapy (ERT) and hematopoietic stem cell transplantation (HSCT) are available for patients with MPS IVA. However, ERT and HSCT do not have enough impact on bone and cartilage lesions in patients with MPS IVA. Penetrating the deficient enzyme into an avascular lesion remains an unmet challenge, and several innovative therapies are under development in a preclinical study. In this review article, we comprehensively describe the current diagnosis, treatment, and management for MPS IVA. We also illustrate developing future therapies focused on the improvement of skeletal dysplasia in MPS IVA.

## 1. Introduction

Mucopolysaccharidosis type IVA (MPS IVA, so-called Morquio syndrome type A) (OMIM 253000) is an autosomal recessive inherited disorder and one of the common lysosomal diseases (LSDs), caused by the deficiency of lysosomal hydrolase, N-acetylglucosamine-6-sulfate sulfatase (GALNS) enzyme (EC 3.1.6.4) [1,2,3,4]. The deficiency of this enzyme accumulates glycosaminoglycans (GAG) such as keratan sulfate (KS) and chondroitin-6-sulfate (C6S) in multiple tissues, mainly bone, cartilage, heart valves, and cornea, leading to devastating skeletal dysplasia with incomplete ossification and successive imbalance of growth [2,5,6].

In 1929, L. Morquio and J. F. Brailsford first reported MPS IVA, independently [7,8]. The prevalence range of MPS IVA was estimated from 1 in 76,000 to 1 in 640,000 births [9,10]. Excessive accumulation of KS and C6S in bone, cartilage, and its extracellular matrix (ECM) causes unique skeletal dysplasia in patients with MPS IVA. Although most patients with MPS IVA generally look healthy at birth, patients often show skeletal deformities within a few years of age. Skeletal dysplasia with short neck and trunk, cervical spinal cord compression, tracheal obstruction, pectus carinatum, laxity of joints, kyphoscoliosis, coxa valga, and genu valgum are common features in a severe form of patients with MPS IVA [5,11,12,13]. MPS IVA patients often become severely handicapped and are forced to become wheelchair bound when they are teenagers. Patients with a severe form die of respiratory problems, cervical spinal cord complications, or heart valve disease in their 20s or 30s if untreated [11,12,13].

Conventional enzyme replacement therapy (ERT) and hematopoietic stem cell transplantation (HSCT) are currently available for patients with MPS IVA; however, there is no definitive therapy for providing a critical impact on bone and cartilage lesions [14,15,16]. ERT and HSCT are based on the principle of cross-correction that lysosomal enzymes are uptaken by deficient recipients’ cells and their lysosomes via the mannose-6-phosphate receptor. ERT for MPS IVA was approved in 2014 by the Food and Drug Administration (FDA) and European Medicines Agency (EMA). However, there are several limitations: (1) weekly infusions for 4–6 hrs, (2) rapid clearance (a short half-life time, 35 min in human, and 2 min in mouse) [17,18], and (3) high price [14,16]. In addition, patients treated with ERT have not shown any remission in skeletal dysplasia or bone pathology [14,15,16,19,20], as observed in MPS IVA mice treated with ERT [18,21]. Thus, there is no proof that the current ERT provides an impact on bone and cartilage lesions in MPS IVA—even after long-term treatment. HSCT could be useful in patients with MPS IVA. Our collaborators and ourselves have experienced 13 cases of MPS IVA patients treated with HSCT, and HSCT for MPS IVA have demonstrated the improvement of pulmonary function, the activity of daily living, bone mineral density, cardiovascular involvement, and laxity of joints, and the reduction of the number of the surgical interventions [22,23,24,25]. However, HSCT often includes several critical issues about (1) finding a matched donor, (2) risks of graft versus host disease (GVHD) and rejection, (3) limited impact on bone and cartilage lesions, and (4) requirement of well-trained staffs and facilities. Most patients with MPS IVA need multiple orthopedic surgeries, including spinal decompression/fusion, leg osteotomies, and hip reconstruction/replacement at an early age. Tracheal reconstructive surgical intervention is often useful to improve airway complications in a severe form of patients [19]. Thus, MPS IVA patients need both symptomatic and supportive therapy in combination with the progression of this disorder.

In this review, we summarize diagnosis, current therapies, and management for MPS IVA. We provide an update on ERT, HSCT, and surgical interventions for patients with MPS IVA and also describe the current situation of developing new therapeutic options, including gene therapy and novel enzyme therapies.

## 2. Diagnosis

### 2.1. Clinical Diagnosis and Phenotype

**Figure 1:**
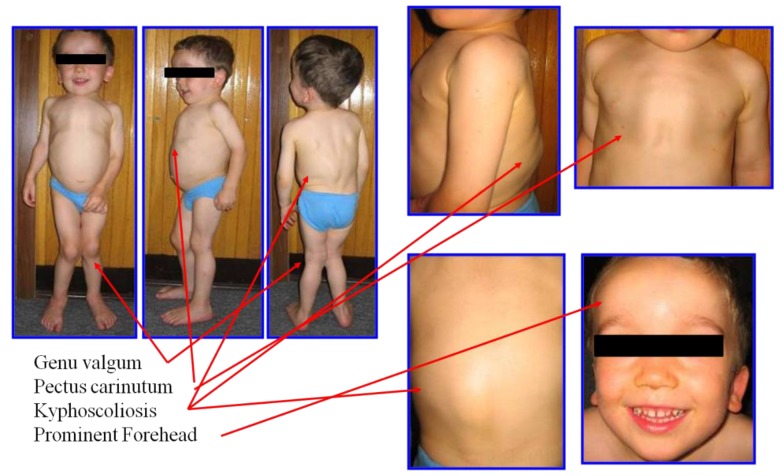
Clinical features of an MPS IVA patient. The patients in this figure had severe form at three years of age and had bone abnormalities of short stature, genu valgum, pectus carinatum, kyphoscoliosis, and prominent forehead. His height is 90 cm with the 50th percentile of male MPS IVA growth chart (adapted from Educational CD for Morquio and permitted by Carol Ann Foundation and Morquio Conference; https://morquioconference.wixsite.com/morquio).

Clinical recognition of devastating skeletal dysplasia is essential to diagnose MPS IVA, combined with the radiographic, genetic, and biochemical tests. Initial clinical signs and symptoms differed depending on the severity of MPS IVA. Most MPS IVA patients usually look healthy at the neonatal period; however, bone abnormalities in the spine can be seen through X-rays even at birth in a severe form of patients with MPS IVA [26]. Patients with attenuated forms have fewer and milder manifestations in bones, and life span is longer than that with more severe form. Skeletal symptoms are found later in childhood or adolescence. Attenuated patients with MPS IVA could survive until more or less 70 years [12]. The first symptoms are associated with the hip region, accompanied by pain and stiffness [27]. In this attenuated form, some clinical signs are related to minor skeletal abnormalities. There is short stature and with the phenotype of osteoporotic pathology [28]. In spite of the severity of MPS IVA, the most common symptoms include genu valgum, coxa valga, kyphoscoliosis, pectus carinatum, wadding gait, spinal cord compression, laxity of joints, and rib flaring (Figure 1).

Airway complications, audiology complications, and dental abnormalities sometimes also provide insights to diagnose MPS IVA. Airway obstruction is a life-threatening issue for this patient, and tracheal obstruction is caused by the imbalance of growth between bones (spine, rib, and manubrium) and vessels or trachea in the thoracic cavity [5,20]. Trachea appears redundant and twisted by images of X-rays, MRI, and CT. This narrowing trachea facilitates and/or worsens respiratory infections and airway obstruction in the lower respiratory tract, causing obstructive lung, loud snoring, look-up to the sky position, shortness of breath, and sleep apnea [29]. 

According to the International Morquio A Registry, less than 50% of MPS IVA patients are diagnosed before 5 years of age. The data from this registry also showed that the most common initial signs are short stature (49.9%), genu valgum (45.1%), kyphosis (44.4%), pectus carinatum (43.6%), and abnormal gait (37.8%) [3,30]. 

### 2.2. GALNS Enzyme and Genetic Diagnosis 

MPS IVA is inherited in an autosomal recessive trait. The human GALNS enzyme was first isolated from human placenta. These tissues were homogenized and processed through precipitation with ammonium sulfate. Once the precipitates were extracted, the extracts were analyzed by chromatographic columns of Concanavalin A Sepharose and DEAE-cellulose chromatography, to isolate the enzyme. After filtration and further isolation by chromatographic columns such as the Ultrogel Aca 34 column, Mono P, and Mono Q, it was possible to isolate this enzyme, subsequently analyzed through SDS-PAGE electrophoresis gels where the two enzyme fractions were observed of 40 and 15 KDa detected by Western blotting. These two polypeptides are interconnected by the disulfide bond [31]. The *GALNS* gene is located on chromosome 16q24.3, which contains 14 exons and 13 introns, spanning approximately 50 kb in length. The *GALNS* gene comprises 1566 bp full-length of cDNA, which encodes 522 amino acids with the 26 amino acid signal peptide [32,33]. In the lysosome, GALNS is stabilized in a complex that includes other enzymes such as *β*-galactosidase, *α* neuraminidase [34], and cathepsin A [26]. Cathepsin A is called “protective protein”, which protects the complex from intralysosomal proteolytic effects. The protection of the enzymes, *β*-galactosidase and GALNS, helps to avoid degradation of these enzymes and consequently promotes degradation of KS [26,35].

Until 2014, a series of studies on patients with MPS IVA conducted in different ethnic populations identified more than 200 different mutations. In MPS IVA as observed in other lysosomal diseases, to determine the correlation between the phenotype and the genotype remains a challenge, due to the high genetic variability and compound heterozygous condition. The previous studies to define the phenotype/genotype correlation have demonstrated the aggravating factors including homozygous mutations, residual enzymatic activity, mutant protein expression study, keratan sulfate level, and in silico model of the GALNS protein. The genotype/phenotype correlation has been established as follows; 85 as severe, 6 as intermediate, and 25 as mild, leaving many mutations without defining its severity [36,37,38,39,40,41,42,43]. In 2016, there were already 327 mutations described, where 242 are missense/nonsense, 32 of splicing, 32 small deletions, 5 small insertions, 2 small indels, 9 gross deletions, 2 gross insertions/duplications, and 3 complex rearrangements [38]. As of December 31, 2019, 362 mutations were reported. To date, the number of mutations of this disease continues to increase [39]. Of all the genetic variations described, the missense point mutation was the most abundant [30]. The three most frequent mutations were represented by missense mutations (p.R386C, p.G301C, and p.I113F). Missense mutations associated with the phenotype were investigated for their effects on the enzyme activity and stability, the levels of KS, the location of mutations in the tertiary structure, and the loci of the altered amino acid residues among the sulfatases [37]. The tertiary structure of GALNS protein showed that three factors provide the severe phenotype; (1) destruction of the hydrophobic core or modification of the packing (p.I113F, p.G301C, p.A351V, etc.), (2) removal or alteration of the salt bridge to destabilize the entire conformation (p.R90W, p.R376Q, etc.), and (3) modification of the active site (p.H166Q, p.G168R, etc.). Reversely, the mutations located on the surface of the GALNS protein are likely to be associated with the attenuated phenotype (p.D60N, p.N204K, p.R259Q, etc.) [37].

Due to the high genetic variation, a genotype/phenotype correlation has been attempted to determine which mutation produces a severe or attenuated phenotype [37,40,41,42,43,44,45,46,47,48,49]. In silico studies were also performed to define the structural change that occurs in the GALNS protein [48]. The genetic information contributes to the clarification of accurate diagnosis and pathology in MPS IVA.

### 2.3. Radiographic Diagnosis

Bone manifestations in the spine and chest are the main clinical symptoms in patients with MPS IVA. During bone development, the ribs are skewered anteriorly and laterally and are very little separated by the paravertebral portions. Kyphoscoliosis with a small and abnormal thoracic cage is a characteristic feature in MPS IVA. All bone manifestations produced are aggravated with age. Another critical feature in this disorder with the accumulation of GAG is odontoid hypoplasia, the lack of ossification in the odontoid process combined with ligamentous laxity, leading to atlantoaxial subluxation and spinal cord compression with consequent cervical myelopathy, quadriparesis, or even death [3,11].

A lack of exercise in this area masks cervical myelopathy, which causes loss of bowel and bladder control, numbness, pain, and weakness, eventually leading to paralysis. At the early stage, the leading cause of mortality in MPS IVA is related to spinal cord compression and the following atlantoaxial, posterior cervical myelopathy. Cervical spinal decompression and fusion surgery is often operated to alleviate neural compression and to stabilize the spine; however, the anesthetic risk must be evaluated due to the difficult airway. If cervical spinal cord compression and cervical instability are untreated, life expectancy in patients with MPS IVA will be between 20–30 years of age [5,50].

When the upper extremities are studied, the irregular epiphyses and widened metaphyses can be commonly seen. The epiphyseal involvement characteristic of MPS IV is exemplified by the tapered irregular distal radius and ulna. The bones are osteopenic with cortical thinning. The mild widening of the diaphysis of the humerus is also visible. With aging, the bone deformity progresses, with tilting of the radial epiphysis towards the ulna. The humerus usually appears shortened later. The hands in time take on a characteristic tilting of the radial epiphysis towards the ulna results from a combination of metaphyseal deformities, hypoplasia of the bones, and degradation of connective tissues near the joint secondary to GAG accumulation [30,51]. The hands have reduced phalanges due to a shortening in the metacarpals as well as irregularities in the carpal bones. The tapering of the proximal portion of metacarpals 2 through 5 and small irregular carpal bones are seen [30,51]. Hyperlaxity of the joints appear mainly at two years of age [2,3].

**Figure 2:**
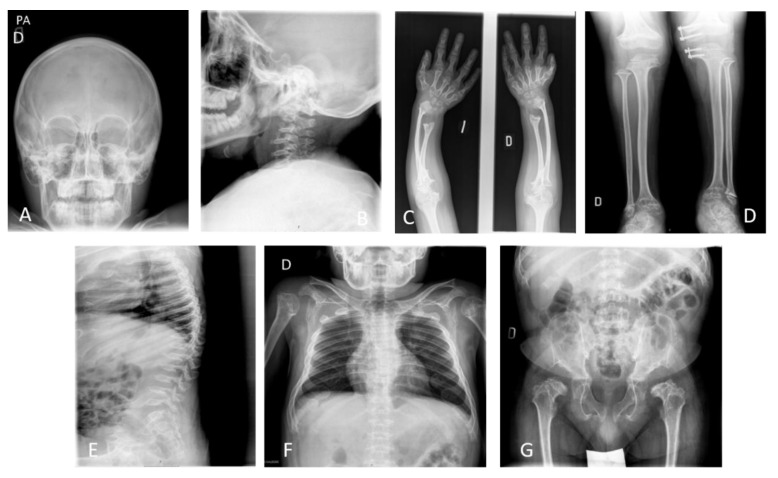
X-ray images of a 17-year-old male patient with MPS IVA. Images show skeletal dysplasia (dysostosis multiplex); (**A**) incomplete ossification and prominent forehead, (**B**) incomplete ossification in odontoid process and subluxation of the atlas secondary to odontoid hypoplasia with platyspondyly of cervical vertebrae, (**C**) cortical thinning and mild widening of the diaphysis of the humerus and tilting of the radial epiphysis towards the ulna producing curvature, (**D**) genu valgum with cortical thinning of tibia and fibula, (**E**) the accentuated dorsal thoracolumbar kypholordosis with the advanced platyspondyly, irregularity, and anterior beaking of vertebral bodies characteristic of MPS IVA and flared ribs, (**F**) abnormal thoracic cage, pectus carinatum and scoliosis with oar shaped ribs: the ribs are wide anteriorly and laterally and overconstricted in their paravertebral portions, (**G**) spondyloepiphyseal dysplastic femoral heads and oblique acetabular roof with coxa valgus deformity and flared iliac wings.

In the study of the hips and lower extremities, multiple bone deformities appear in the pelvic bones, including spondyloepiphyseal dysplastic femoral heads and oblique acetabular roof with coxa valgus deformity and flared iliac wings. The most common lower extremity deformities are genu valgum (knock knees) [12,52,53]. A skew foot posture is seen for both legs as well as an increased sandal gap between toes 1 and 2, as shown in Figure 2.

### 2.4. Biochemical Diagnosis 

#### 2.4.1. GAG Level in Urine

The diagnosis for MPS IVA can be completed with unique clinical features and biochemical analyses in blood and urine samples. Multiple techniques have conducted studies on the measurement of total urinary GAGs. A universal method is through a chemical reaction that manifests itself in a colorimetric manner, where the detection of GAG is due to the union of nonspecific across disulfide bridges with the dimethylmethylene blue (DMB) [54]. This technique has the advantage of being fast and economical; however, this method is less used because of many false positives, especially in the samples of MPS IVA patients [54,55,56,57]. Urine GAG levels did not clearly distinguish the MPS IVA patients from normal individuals. Approximately 20% of MPS IVA patients overlapped with the normal range. DMB method can not be applied to blood samples since the blood samples include an abundance of proteins to interfere in the reaction.

#### 2.4.2. KS Level

The levels of blood and urine KS are age-dependent. The ELISA method was first developed for the detection of KS in blood and urine [56]. The method separated the age-matched control samples from the samples in MPS IVA patients. Blood KS levels in MPS IVA patients were two to eight folds higher than those in age-matched controls. The blood KS level varied with age and clinical severity. Blood KS levels in both MPS IVA and controls peaked between 5 and 10 years of age. Blood levels in severe MPS IVA were higher than in the milder form. In contrast to blood, urine KS levels in both MPS IVA and controls peaked between 1 and 5 years of age, and after that declined with age. Urine KS levels for MPS IVA patients and normal controls were different at any age. After 5 years of age, urine KS levels in MPS IVA began to decrease gradually. This decrease was the most remarkable in the patient group between ages 5 and 10 years of age and 10 and 15 years of age. After 15 years of age, the KS excretion continued to decline and reached a plateau after 20 years of age. The excretion of urine KS was significantly different between MPS IVA patients and normal controls even after age 20 years. Although the degree of decline in the urine KS concentration proportional to age paralleled that of blood KS concentration, urine KS level remained higher than the control.

Another technique to measure blood and urinary KS is by liquid chromatography tandem mass spectrometry (LC-MS/MS) [58,59,60,61,62]. Blood and urine KS measurements in patients with MPS IVA were age-dependent and higher than age-matched healthy controls [60,61]. We observed a moderate correlation between urine KS measurements and a weak relationship between blood KS measurements by ELISA and LC-MS/MS methods in patients [61]. The difference between KS measurements assayed by LC-MS/MS and ELISA was more significant in controls than in patients. A moderate correlation between blood and urine KS measurements in the same individual was observed. These findings indicate that both methods to measure blood and urine KS are suitable for diagnosis, monitoring therapies, and longitudinal assessment of the disease course in MPS IVA, but the LC-MS/MS method measures over 10 times more KS present in body fluids. This type of analytical tool allows more extensive studies to detect KS in different biological specimens (blood, urine, dried blood spots, tissues, etc.) and applies to newborn screening with high sensitivity and specificity, which makes it feasible to reduce the false positive and negative in the diagnosis [63,64,65,66,67,68,69]. Although the determination for the KS in urine and blood leads to precise diagnosis in MPS IVA, more biomarkers, including pro-inflammatory factors, are required for the prognosis, therapeutic efficacy, and clarification of pathogenesis with the development of advanced therapies [70,71].

#### 2.4.3. C6S Level 

C6S is accumulated in systemic tissues, especially growth plates, aorta, and cornea in patients with MPS IVA [72,73,74]. Shimada et al. [75] established a quantitative method for C6S in human blood and urine using LC-MS/MS by separating C6S from other CS disaccharides. The levels of C6S in blood and urine are age-dependent similar to that in KS. This novel method showed that blood and urinary C6S combined with KS level can be a useful biomarker not only to screen and diagnose MPS IVA but also to assess the clinical severity and therapeutic efficacy.

#### 2.4.4. Enzyme Assay 

The final diagnosis of MPS IVA is confirmed by the GALNS enzyme assay. This biochemical technique widely used determines the enzyme deficiency in biological samples, including blood (plasma or serum), leukocytes, dried blood spot (DBS), and skin fibroblasts [76,77]. It is also possible to perform prenatal diagnosis by using amniotic fluid, dissected chorionic villi, and cultured chorionic villus cell [78,79,80]. The residual enzyme activities in some of the mutations correlate with clinical severity [36,37,38,39,40,41,42,43,44,45,46,47,48,49], especially if the residual activity is high (associated with the attenuated phenotype; p.N204K, p.T312S, etc.); however, the absence or trace of enzyme activity is observed even in the attenuated-associated mutations. 

To date, the most used techniques for the enzyme assay are through fluorometric technique [81,82] and LC-MS/MS [83]. LC-MS/MS technology provides more precise and sensitive quantification; however, the enzyme activity in DBS samples is unstable, and therefore, the DBS sample must be kept carefully during the storage and transport to the laboratory to reduce false positives. It is preferable to assay several lysosomal enzymes as a reference to rule out other LSDs. 

One of the main problems in MPS IVA is to establish a rapid and accurate diagnosis for these patients since skeletal dysplasia is progressing rapidly. If these patients are diagnosed late, it is too late to correct bone deformation in timely management. In recent years, new programs for establishing early diagnosis before an irreversible and/or serious condition have been proposed. One example is a project that emerged 5 years ago in Spain. This project, which covers the entire country, aims to publicize the existence and awareness of MPS disorders. The project has been coordinated with experienced physicians. When the physicians see patients with the clinical features whom they suspect MPS, they can acquire the samples from patients and send them to reference laboratories. The required samples are urine and blood from the patients that can be collected on chromatographic paper, regardless of age, race, or sex. To make a rapid and precise diagnosis, the first test is the DMB technique where the total urinary GAG is measured. In the case of finding the elevation of GAGs, capillary electrophoresis of the urine sample is performed to determine the elevated specific GAGs. The study is completed by performing an enzyme activity test to diagnose the type of MPS. In the first 2 years alone, 8 cases were diagnosed as MPS, of which 2 were diagnosed as MPS IVA [84]. In some countries, instead of the DMB method, LC-MS/MS is used for GAG analysis, followed by the enzyme activity assay.

## 3. Treatment and Management

### 3.1. ERT

ERT for MPS IVA (recombinant human GALNS, which is referred to elosulfase alfa; trade name Vimizim) was approved by FDA and EMA in 2014. Currently, this drug is used in the United States, European countries, and other developed countries. Results of clinical trials in a short period showed that elosulfase alfa was well-tolerated and provided improvement of endurance, stabilized forced vital capacity (FVC), forced expiratory volume in 1s (FEV1), and left ventricular mass index [85,86,87,88,89,90,91]. While urinary KS level was significantly decreased during receiving ERT, the blood KS level was not changed compared with that in untreated patients [92]. In phase III clinical trial, all MPS IVA patients treated with ERT had anti-drug antibody (ADA) and immunoglobulin E (IgE); however, there was no correlation between high anti-drug total antibody (TAb) titer and worsening efficacy or safety profile [93]. A small number of treated MPS IVA patients tested positive for ERT-specific IgE, and its positivity was not consistently associated with either the occurrence or severity of anaphylaxis [94]. Although there were reports about the positive impact of measurements such as FVC and FEV1 in pulmonary function test [85,86], Kenth et al. showed a global decline in static spirometry values of all subjects treated with ERT after a long-term follow-up [95]. This long-term study demonstrated that a global reduction in spirometry variables and improvement post adenotonsillectomy, while the overall results being a reduction in pulmonary function, suggesting that non-invasive ventilation and adenotonsillectomy should be more effective in the patients with ERT, either improving lung function or attenuating aggravation of symptoms. We have experienced tracheal reconstitution surgery in MPS IVA patients treated with ERT since 2015 (See the “Management of tracheal obstruction” chapter) and showed that the accumulation of storage material was observed in surgical remnants of trachea from these patients [19]. After this tracheal surgery, the obstructive airway was resolved, and their pulmonary function and activity of daily living were markedly improved. Overall, these studies showed that conventional ERT for MPS IVA has little impact on airway obstruction in spite of long-term treatment.

MPS IVA is characterized by devastating skeletal dysplasia, and therefore, the most expected therapeutic effect for MPS IVA provides an impact on bone and cartilage lesions. To date, there has been no report on the effect of ERT on the resolution of these skeletal lesions in patients with MPS IVA. We showed that chondrocytes and ECM derived from these patients, who had received ERT for 6 to 30 months, showed no reduction of vacuoles in bone pathology [14,15]. These pathological features are consistent with the fact that a frequency of need for orthopedic surgery has not been reduced after receiving ERT [96]. 

Short stature is the most critical and measurable feature for skeletal dysplasia in MPS IVA. We also assessed cross-sectional and longitudinal data of heights and weights collected from 128 MPS IVA patients, compared with the growth charts of MPS IVA [20]. Except attenuated patients, males and females patients with severe forms of MPS IVA tarting ERT even before 5 years of age failed to show significant improvement of growth. Treated and untreated patients reached their final heights by approximately 10 years of age. Patients treated with ERT still showed a declined pubertal growth spurt, which contributes to marked short stature as observed in untreated patients. These results are in line with those of Cao et al., which indicated that early ERT starting at 21 months did not improve the bone growth in a severe patient with MPS IVA, as determined after the 30 month-long term treatment [87]. Patients treated with ERT did not show any significant increase in the growth in any age group, compared with the growth charts for untreated patients. 

Overall, until now, there has been no proof that the current ERT provides the impact on bone lesions and consequent skeletal dysplasia in MPS IVA patients. Therefore, to improve the existing and/or future skeletal issues remains a unmet challenge in MPS IVA.

Under these circumstances, the issue of approval of current ERT occurred in some countries. ERT for MPS IVA was made available for a limited period at a discounted price in the UK and Canada. In addition, some European countries (The Netherland and Belgium) and Australia decided to stop reimbursement of elosulfase alfa due to the cost-benefit imbalance of this ERT ($500,000/year/25 kg patient) [4,16]. 

To overcome the limited impact on bone and cartilage lesions of MPS IVA patients, the bone-targeting strategy should be required to enhance the therapeutic efficacy on their bone deformities. We have developed the GALNS enzyme tagged with the acidic amino acid peptide as bone-targeting ERT [21]. This modified enzyme had a markedly prolonged clearance from the circulation, leading to 10–20 times higher level of the enzyme activity in blood than those of the native enzyme in an MPS IVA mice. Then, the accumulation of storage materials in the growth plate and the articular cartilage in mice treated with the bone-targeting enzyme was markedly reduced, compared with that of mice treated with the native enzyme. Therefore, bone-targeting ERT provides a more impact to the bone lesions of this model mouse, and this modified enzyme can be a potential treatment for MPS IVA.

### 3.2. HSCT

The first successful case report of HSCT in MPS IVA was published in 2014 [22]. Chinen et al. reported a male patient with MPS IVA who underwent allogeneic bone marrow transplantation (BMT) at age 15 years 8 months. At 5 years post-HSCT, the recipient GALNS activity in lymphocytes had matched the level measured in the donor, accompanied by several clinical improvements such as pulmonary function and increased bone mineral density (BMD). Although his height was not changed at 106 cm, pulmonary function was well-stabilized during 9 years post-BMT. BMD in the lumbar vertebrae (L2-L4) was also increased by 50% at one year post-BMT. 

In 2016, Yabe et al. reported allogeneic HSCT cases in 4 patients with MPS IVA, including a patient in the case study reported by Chinen et al. [22,23]. Transplantation was successful in all 4 cases without any serious GVHD. All of the patients achieved complete engraftment, and the GALNS enzyme activity of the recipient’s lymphocytes in all cases reached into the donor’s level (2 patients displayed normal enzyme activity). HSCT improved the clinical progression of MPSIVA in these patients. The scores of the activity of daily living (ADL) in MPS IVA patients treated with HSCT were better than in age-matched untreated patients and remained elevated thereafter. The patient who had been treated at the earliest age (4-year-old) had the highest ADL score, indicating that early treatment could have a more significant impact on bone growth. None of these treated MPS IVA patients developed serious valvular heart disease. Patients with a severe phenotype often undergo multiple orthopedic surgeries such as spinal cervical decompression and fusion, leg osteotomy, knee correction, and hip correction or replacement through life. However, 3 out of 4 patients have had no surgical intervention post-HSCT. Only one patient underwent a bilateral osteotomy after transplantation. Although there was a definite improvement in walking, stair climbing, endurance, and hand movement in all four cases after HSCT, HSCT has a limited impact on bone growth of MPS IVA since the transplantation was conducted after the bone growth has stopped or nearly stopped. 

In 2016, Wang et al. reported the other 4 patients with MPS IVA treated with HSCT in China [24]. Improvements observed in one of these MPS IVA patients included remissions in hepatosplenomegaly, joint hypermobility, upper airway obstruction, and recurrent otitis media, and slight improvements in height and thoracic deformity. After HSCT, the patient’s spinal cord compression was also stabilized. The patient underwent surgical intervention for genu valgum at 1 year after HSCT. To date, HSCT had been conducted to treat 9 patients with MPS IVA (median age, 3 years; range, 1.58 years) at Shanghai Children’s Medical Center [25]. All MPS IVA patients with HSCT have achieved complete donor chimerism with normal enzyme activity level without any serious complication, and all were alive with significantly reduced joint hypermobility. Two patients underwent surgical interventions for spinal cord compression, genu valgum, and hip dislocation.

Overall, the long-term follow-up of HSCT in MPS IVA patients showed that this cell-based therapy achieved the donor’s GALNS activity level, improved pulmonary function, BMD and ADL, and reduced the frequency of surgical intervention, suggesting that HSCT could be a useful supportive treatment option for patients with MPS IVA.

### 3.3. Orthopedic Surgery

Patients with MPS IVA often develop kyphoscoliosis and thoracic deformity in the first year. Within a few years after birth, over 70% of MPS IVA patients show skeletal abnormalities such as pectus carinatum, kyphoscoliosis, laxity of joints, and genu valgum [11,97]. Currently, approved therapies such as ERT and HSCT do not have enough impact on established skeletal dysplasia in MPS IVA patients. MPS IVA patients often require multiple orthopedic surgical interventions in the upper cervical spine and lower extremities until their mean age of 10 years [11].

Cervical spine surgery: Cervical instability in the neck and the upper spinal cord compression among children with MPS IVA are caused by odontoid dysplasia, incomplete ossification, and ligamentous laxity of the anterior and posterior atlas, leading to quadriplegia, chronic myelopathy and sudden death by respiratory failure [11,12,13,30,98,99]. Upper cervical spine decompression with fusion from occiput to C2 is recommended to alleviate spinal cord compression in patients with MPS IVA. Computed tomography (CT) and magnetic resonance imaging (MRI), as well as X-ray images, are standard procedures to evaluate compression and instability in the upper cervical spine of general MPS patients, including MPS IVA. Imaging tests, including MRI and radiographs before 2 years of age are required routinely. Patients with MPS IVA should be followed annually to determine whether orthopedic surgery is indicative. Thus, early diagnosis of MPS IVA and management of cervical spine compression, instability, and spinal stenosis are required to prevent myelopathy and other complications [100,101]. However, in a long-term follow-up study, distal junction instability is another major issue after cervical surgery in MPS IVA patients, and an additional surgical intervention in the lower cervicothoracic spine remains necessary in 40% of patients [102]. 

Low extremity surgery: Progressive coxa valga, genu valgum, and ankle valgus are notable features of the lower extremity in patients with MPS IVA [103] (Figure 2). These deformities are developed enough severe to require surgical interventions in MPS IVA children [52]. Hips appear either normal or partially dislocated as early as the second year of life in the severe form of MPS IVA patients. Hip dysplasia and progressive subluxation are universally seen in MPS IVA; however, they are not determined, despite the presence of a significant cartilaginous analog on MRI and arthrogram [52]. MPS IVA children with severe form show small capital femoral epiphyses, which are progressively flattened, fragmented with growth, and finally lost in adulthood. Hip subluxation and associated pain often force MPS IVA patients to become wheel-chair bound as teenagers, if they do not receive any orthopedic surgery [104]. Hip reconstructive surgery such as shelf acetabuloplasty and varus derotation osteotomy has been recommended at the early life of MPS IVA. These orthopedic surgeries reduce hip pain and improve its movement; however, the surgery is a challenge in patients with MPS IVA due to a deformed acetabulum [53]. Patients with MPS IVA are prone to have subluxation after shelf acetabuloplasty and varus derotation osteotomy [52]. Hip replacement surgery is sometimes required in adult patients with MPS IVA, who have severe arthritis and pain [13,104].

Patients with MPS IVA also have lower extremity impairments such as genu valgum, knee flexion, and external tibial torsion, and these deformities need orthopedic surgeries to improve function [13,104]. Knee deformity is the most common feature and is observed in children with MPS IVA at 3 years of age. Growth guidance techniques (two-hole growth modulation plates) may be a useful option to correct genu valgum, particularly in smaller children, as they allow for more reliable fixation in the small cartilaginous epiphyses if patients have sufficient growth plates [52,105]. Osteotomy of the proximal tibia or distal femur may be required in patients with limited remaining growth. However, it is difficult to reverse established severe genu valgum to the patient’s preoperative level of function [53]. Thus, genu valgum-related care should be started before the progression of the deformity, and patients may require surgical interventions as young as 4 years of age [53]. Repeated surgeries for a knee deformity are often needed during the bone growth in MPS IVA children.

### 3.4. Management of Tracheal Obstruction

**Figure 3:**
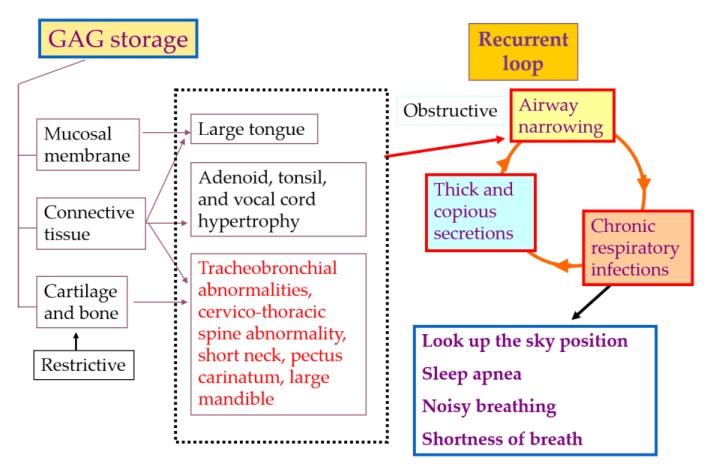
Pathophysiology of airway compromise in MPS IVA patients (adapted from Educational CD for Morquio and permitted by Carol Ann Foundation and Morquio Conference; https://morquioconference.wixsite.com/morquio).

Patients with a severe type of MPS IVA sometimes do not survive beyond 20 to 30 years of age since they often have respiratory compromise or spinal cord compression. Tracheal obstruction in patients with MPS IVA is one of the life-threatening issues and provides high risks of death because of severe sleep apnea and related complications [5,50]. This obstruction of patients with MPS IVA can be caused by multiple factors: (1) disproportionate growth of the trachea, brachiocephalic artery, neck, and chest cavity (redundant trachea), (2) destructive nature of GAG accumulation in the airways, and (3) a severe pectus carinatum narrowing of the thoracic inlet and thoracic cavity [19,87] (Figure 3). The disproportionate length of the trachea and vessels in the thoracic cavity in comparison with short neck and pectus carinatum can provide the problem of twisting trachea and compressed trachea by the brachiocephalic artery. While the trachea does not have the same growth restriction as bone, the trachea will grow relative to the adult-size and be redundant in restricted space. Multiple imaging techniques such as angio-CT, X-ray, and MRI often help reveal the narrowing airways in MPS IVA patients. However, relatively little attention has been paid to the monitoring of tracheal obstruction progression [19].

It remains unknown whether ERT can prevent tracheal obstruction of patients with MPS IVA. Currently, ERT has no impact on bone growth in this patient—even if ERT starts under 5 years of age [20,87]. Because of this situation, no matter how well active enzyme penetrates target organs and works to improve trachea, the trachea will never have enough room to extend normally [106]. Thus, it is likely that surgical intervention will continue to be required to correct airway obstruction. Although tracheostomy is currently a standard surgical intervention to improve airway obstruction in MPS IVA patients, patients lose their ability to communicate through speech after the procedure because the tracheostomy tube blocks the air from passing through the voice box form the lungs [106]. As of the end of 2019, 10 of 12 cases underwent a new surgical intervention to rescue severe tracheal obstruction successfully (8 patients were under ERT over 1 to 5 years). As this surgical procedure, the brachiocephalic artery was repositioned, moving it rightward on the ascending aorta so that it rested parallel to the trachea instead of crossing in front of it. Then, the redundant trachea was removed, and the remaining ends were brought together and connected [19,106]. Patients who successfully underwent this tracheal reconstructive surgery showed marked improvement of their respiratory status and activity of daily living immediately; however, it is noteworthy that the surgical process provides a high risk because of difficult airway—especially in the presence of spinal cord compression. The well-trained multi-discrepancy team should be required to the success of this tracheal reconstructive surgery.

### 3.5. Future Therapies

Several treatment candidates for MPS IVA are underway in preclinical studies using animal models. In this chapter, we describe the current status of development of these treatments, including substrate degradation enzyme therapy, adeno-associated virus (AAV) gene therapy, nanomedicine, and pharmacological chaperone therapy (Table 1).

#### 3.5.1. Substrate Degradation Enzyme Therapy (SDET)

Lysosomal hydrolases are only active in the acidic pH, which is maintained within lysosomes, indicating that the conventional ERT target is to avoid the accumulation of GAG in this organelle. However, recent studies have shown that not only accumulated GAG within cells but that in the neighboring ECM provide an important role in developing abnormal chondrogenesis and endochondral ossification [107,108,109,110]. We have focused on a KS hydrolase, endo-*β*-*N*-acetylglucosaminidase from *Bacillus circulans*, which have a characterization of thermostability. Thus, this enzyme is a so-called thermostable keratanase [111]. We purified thermostable keratanase and assessed the effect of this glycosidase on blood KS level and bone pathology using MPS IVA mice. After a single administration of 2 U/kg (= 0.2 mg/kg) of this enzyme at 8 weeks of age via intravenous injection, the level of blood KS was significantly decreased to normal range level, and this suppression was maintained at least up to 12 weeks of age. We administered 2 U/kg of thermostable keratanase to MPS IVA mice every fourth week for 12 weeks (total of 3 times) to newborns. After three times injection, bone pathology was markedly improved when the administration of this enzyme started from the neonatal period, compared with untreated MPS IVA mice. We termed this novel therapy as “*Substrate degradation enzyme therapy* (SDET)” [112]. Dose-dependent study and immunosuppression strategy will be required to develop this therapy for moving forward to clinical trials for MPS IVA.

#### 3.5.2. Gene Therapy

Gene therapy has been a rapidly developing treatment modality for inborn errors of metabolism, and several clinical studies have investigated its use for the treatment of the MPS. Currently, gene therapies are being investigated in animal and human trials for various MPS subtypes with reported success in achieving transgene expression via lentivirus [113,114], retrovirus [115], and AAV [113,116,117] mediated gene therapy. AAV is an advantageous vector option due to its non-pathogenicity and efficient transduction potential, as demonstrated in several in vitro and in vivo animal models [117,118]. Lentiviral and AAV-mediated gene therapies are currently under investigation in clinical trials for MPS subtypes I, II, III, and VI. For the treatment of MPS IVA, gene therapy is relatively new, with only a few pre-clinical studies investigating its safety and efficacy [12,119,120,121,122]; however, its potential as a potent therapeutic agent is evident, and the continuous investigation is necessary to optimize this therapy.

AAV was initially shown in 2008 to successfully transduce diseased MPS IVA cells in vitro and achieve increased GALNS activity [123]. Alméciga-Díaz et al. then developed an AAV2 vector containing CMV, AAT, or EF1 promoters and GALNS cDNA and evaluated enzyme activity in HEK293, human MPS IVA fibroblasts, and murine MPS IVA chondrocytes with co-expression of sulfatase modifying factor 1 gene (SUMF1) and found significantly increased levels of enzyme activity in treated cohorts [119]. Recently, an in vivo murine model was developed by Tomatsu et al. to investigate the ability of AAV to target bone lesions. Modifications were made to the AAV2 vector by adding an acidic amino acid sequence (aspartate octapeptide D8) to the AAV capsid to increase its affinity for hydroxyapatite, a key component of bone [122]. After the administration of the modified and unmodified AAV2 containing chicken *β*-actin (CBA) promoter and hGALNS, the enzyme activity within the bone of the modified vector cohort significantly increased to 42% of wild-type levels, compared to 4.8% in the unmodified cohort [122]. The results, which demonstrate successful targeting of bone—a notably a hard-to-reach tissue due to poor vascularization—can be achieved with modification of the AAV vector. AAV therapy remains a work in progress, and the full potential of this therapy is limited by the immune response generated against the capsid, transgene product, and transduced cells [124,125]. Methods of overcoming the immune response include novel serotype generation [126], promoter design and liver targeting [127,128], exosome coupled AAV vectors [129,130], and several others currently being investigated.

In its translation from pre-clinical to clinical trials, gene therapy for MPS has demonstrated therapeutic efficacy with the initial success as phase I/II trials are underway. While no clinical trial is currently developed for MPS IVA, analysis of other MPS subtype trials may be beneficial due to disease homology. Regenxbio (Rockville, MD, USA) has developed an AAV9-mediated gene therapy for the treatment of MPS I and II, RGX-111 and RGX-121, respectively. In December 2019, Regenxbio announced an update to their RGX-121 clinical trial, revealing that significant cerebrospinal fluid (CSF) substrate reduction had been achieved in Cohort 1 (*n* = 3) with patient 1 exhibiting a 23.7% reduction from baseline to week 8 and a 43.6% reduction from baseline to week 48; patient 2 showing a 30.9% reduction from baseline to week 8; and patient 3 achieving a 41.6% reduction from baseline to week 8 [131]. It was also reported that no drug-related serious adverse events occurred, and that neurocognitive development is stable in the two patients treated for greater than 24 weeks [131]. Abeona Therapeutics (New York, NY, USA) who developed an AAV9 vector for the treatment of MPS IIIA announced in July 2019 that continued reduction in CSF heparan sulfate (HS) was observed in all three cohorts with the highest dose cohort achieving the lower threshold of detectable HS [132]. Additionally, mental development in the three youngest patients is progressing at a normal age equivalent. Lysogene (Neuilly-sur-Seine, France) is currently investigating the use of AAVrh10 (LYS-SAF302) in the treatment of MPS IIIA with the first patients dosed in February of 2019. They recently published data on LYS-SAF302 in large animal models, which confirmed the efficacy of the drug, showing greater than 20% enzyme activity in 78% of dogs and 97% of monkeys [133]. Apart from the AAV-mediated treatment model, Orchard Therapeutics (London, UK) has started a Phase I/II ex vivo gene therapy using autologous CD34^+^ cells transfected with hSGSH-containing lentiviruses [134]. Pre-clinical data for this treatment modality demonstrated competency in meeting expected safety profiles and an effective transfection goal (2–5 copies/cell) [135]. This treatment model is the first of its kind in MPS clinical trials, and the preliminary results are highly anticipated.

While these clinical trials show promise for the development of an MPS IVA clinical model, the ability to target bone lesions via AAV or other vectors remains a limitation of potential treatment. While modifying the AAV capsid demonstrated a greater affinity for bone [122], other methods such as serotype and promoter optimization may be necessary to deliver vectors to difficult-to-reach tissues. Lee et al. performed a series of experiments investigating various AAV serotypes, both natural and engineered, in combination with two different bone-specific promoters Sp7 and Col2.3 to assess their ability to transduce human osteoblasts in vitro and murine models in vivo [136]. The serotype assay showed transduction efficacy ranging from 14% to 56% in human fetal osteoblast cells and, in mice, AAV8 with Sp7 promoter showed the highest affinity for osteocytes in fractured bones [136]. Strategies such as capsid modification to increase affinity for bone structural components, in addition to serotype and promoter selection for optimal transduction, may yield greater transduction results and the combination of various techniques should be further investigated.

With the current state of gene therapy for the treatment of MPS, there lie promising results in the pre-clinical and clinical data. In vivo and in vitro studies investigating gene therapy for the treatment of MPS IVA have laid the framework for the development of a clinical model for the treatment of this MPS subtype. The first-generation AAV gene therapy in clinical practice will grant a clearer understanding of the effects of these technologies on human patients, and from their improvements to immune response reduction or target tissue tropism can be further developed. The future for MPS IVA is promising as improvements are continually made to gene therapy options, demonstrating its safety and efficacy as a treatment.

#### 3.5.3. Nanomedicine

Although the term nanomedicine is not used until the first decade of 2000, the use of colloidal gold has been known for a long time. However, the true development of nanomedicine has started in the late 1970s and early 1980s [137]. 

The nanomedicine uses different nano-sized tools for the diagnosis, prevention, and disease treatment to improve quality of life. Until now, different nanostructures and materials have been applied to therapeutics. Nanomedicine includes a great variety of structures including particles as polymeric nanoparticles, metallic nanoparticles, nanocrystal, nanosuspensions, nanocomplexes, polymer-protein conjugates, quatum dots, dendrimers, mesoporous nanoparticles, lipid nanoparticles or nanostructured lipid carriers; vesicles as liposomes, exosomes, polymersomes or niosomes and emulsions as microemulsions or nanoemulsions.

In recent years, nanomedicine has emerged as an alternative or combination for the treatment of LSDs to improve the effectiveness and the safety of ERT or gene therapy [138,139]. Nanomaterials present a great potential for their use in the treatment of LSDs by integrating the therapeutic molecules (i.e., enzymes, gene, etc.), maximizing their efficacy and reducing dose and toxicity and providing drug targeting, controlled and site-specific release improved transport across biological barriers and promoting a differential distribution within the body (e.g., bone, brain, etc.) [140,141]. One of the advantages of nanomedicine is the capacity of some nanostructures to cross the biological barrier as the blood-brain barrier [142,143,144,145], where the main barrier for the effective treatment of MPS was due to neurological complications. In 2019, Donida et al. reported the capacity of nanoparticles to cross BBB feasibly [146]. Another advantage of nanostructures compared with native enzymes is the enhanced cellular uptake due to the endocytosis internalization processes used by nanostructures [142,147] independent of the mannose-6-phosphate receptors (M6PR) used by recombinant enzymes, since the M6PR are easily saturated [148,149,150,151]. Additionally, the clathrin-mediated internalization pathway used by some nanostructures as nanostructured lipid carriers produces the internalized nanostructure trapped inside the endosomal/lysosomal vesicles, resulting in its degradation by lysosomal enzymes and in the subsequent release of the immobilized material (i.e., ERT enzyme) [142].

**Figure 4:**
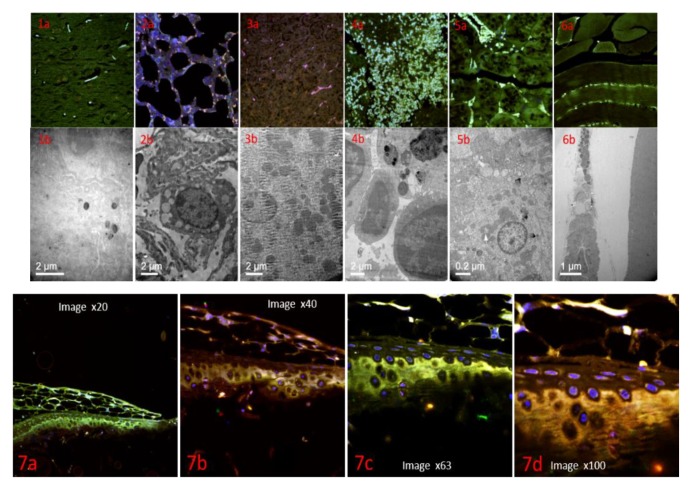
Distribution of nanostructured lipid carriers containing GALNS in mouse tissues. Images different tissues in wild type mouse, (**1a**) confocal microscopy brain tissue, (**1b**) electronic microscopy nanoparticles inside of neuron cell, (**2a**) confocal microscopy lung tissue, (**2b**) electronic microscopy nanoparticles inside of pneumocyte cell, (**3a**) confocal microscopy liver tissue, (**3b**) electronic microscopy nanoparticles inside of hepatocyte cells, (**4a**) confocal microscopy spleen tissue, (**4b**) electronic microscopy nanoparticles inside of macrophages cells, (**5a**) confocal microscopy kidney tissue, (**5b**) confocal microscopy renal tubule cells, (**6a**) confocal microscopy muscle tissue, (**6b**) confocal microscopy fibroblast cell, (**7**) images of mouse cartilage of mice at different zoom, showing that the cell inside of the cartilage contains nanoparticles (pink color) (**7a**) picture of cartilage ×20 zoom, (**7b**) picture of cartilage ×40 zoom, (**7c**) picture of cartilage ×63 zoom, (**7d**) picture of cartilage ×100 zoom.

In 2019, Alvarez et al. studied the effects of protein expression levels in MPS IVA fibroblasts treated with free elosulfase alfa or immobilized in nanostructured lipid carriers (NLC). Enzyme immobilized in NLC showed a greater and more effective cellular internalization improving the traffic of molecules between different organelles in the cytoplasm. The communication of the information between lysosomes and other organics is less efficient, due to the malfunction of lysosomes, As with the amino acids that are recovered from the lysosome and are reused by mitochondria [152], transferrin transport is also affected [153] in autophagy phenomena [154]. There are also problems with the pathosystem endosome [155] due to cholesterol storage in the membrane of that endosome. This cholesterol reserve blocks a complex necessity to direct the lysosome [155]. Regarding of MPS IVA, there has been little study on the effect of nanomedicine [142,156]. In 2019, Alvarez et al. developed a NLC containing elosulfase alfa and studied its efficacy using in vitro cellular models and its in vivo biodistribution in mice. The solid formulation easily re-dispersed in injectable medium has been devised to protect and stabilize the enzyme. The results showed the capacity of the NLC to internalize cells and to reach lysosomes releasing the enzyme. Experiments developed using pathological tissues showed the ability of the nanostructures to release the GALNS enzyme in target cells and to degenerate accumulated GAGs in affected cells. Additionally, the in vivo tissue distribution after intravenous administration into mice showed the capacity of the NLC to reach hard-to-accessible organs (i.e., brain, cartilage, bone, etc.) (Figure 4) [142]. 

Nanomedicine appears an excellent tool to improve the efficacy of ERT due to the following reasons; (i) to protect the enzymes and avoid molecular recognition by the immune system, preventing the selective production of antibodies, (ii) to promote the cell uptake and the drug delivery inside in the lysosomes, and (iii) to promote the crossing of the biological barrier better than the enzyme alone.

#### 3.5.4. Pharmacological Chaperone Therapy

According to several studies on MPS, most of the mutations on lysosomal enzymes may affect protein processing, folding, glycosylation, and stability [37,157]. Based on these findings, the use of small molecules—called pharmacological chaperones—can improve protein misfolding, intracellular trafficking of the enzyme, abnormal protein aggregation and enhanced ER stress [158]. Pharmacological chaperone therapy has been evaluated in LSD [159,160,161] including MPS II [162] and MPS IIIC [160]. The advantage of pharmacological chaperone therapy compared with ERT is (1) wide distribution of the drugs into multiple tissues including the central nervous system, (2) availability of oral administration, and (3) low possibility of immunogenicity. However, there is still unknown information about; (1) intracellular distribution of the drugs, (2) off-target side effect, (3) inhibition of the activity on the enzyme, and (4) availability on a variety of mutations [163]. Recently, Alméciga-Díaz et al. showed that two potential GALNS pharmacological chaperones for MPS IVA—ezetimibe and pranlukast—were identified by molecular docking-based virtual screening [164]. Both chaperones significantly increased GALNS activity in MPS IVA fibroblast cells carrying p.R61W, p.W405_T406del, and p.A393S mutations. Combined treatment of recombinant GALNS with ezetimibe or pranlukast also provided an additive effect in this fibroblast. Further studies should be required to evaluate the impact of these compounds on bone and cartilage lesions using animal models.

## 4. Conclusions

MPS IVA is characterized by severe systemic skeletal dysplasia, subsequent airway obstruction, spinal cord compression, and cardiovascular disease, which are often life-threatening issues for patients with this disorder. However, conventional ERT provides a limited impact on bone and cartilage lesions of patients with MPS IVA [14,15,16], even if it started at an early stage. Although several studies showed that HSCT may have effects on bone, there is no proof to improve bone growth after long-term observation of treatment with HSCT starting at over 4 years of age [22,23,24,25]. Due to avascular characteristics of bone lesions, secreted or infused enzyme in circulation is difficult to penetrate cartilage cells of growth plates and articular discs. An advantage of gene therapy in MPS IVA is to provide sustained high levels of the enzyme in circulation. If supraphysiological enzyme activity levels are obtained, enough enzyme should be delivered in bone and cartilage lesions. In addition, the bone-targeting method is another option to improve the delivery of enough enzymes into bone and cartilage lesions. Our developing bone-targeting method using oligopeptide showed the potential for improving skeletal abnormalities in MPS IVA [21,122], and this bone-targeting strategy combined with gene therapy and new ERT should be new candidates for this disorder. Early diagnosis is necessary to obtain enough effects of therapies and prevent the progression of this disease since the progression of skeletal dysplasia in MPS IVA starts from neonatal periods. We have established a highly sensitive and specific quantitative method to measure GAG by LC-MS/MS, and newborn screening using our method should help early diagnosis for MPS patients, including MPS IVA [65,66,75]. Therefore, both early diagnosis and effective therapy for MPS IVA are required to cure skeletal dysplasia of patients with MPS IVA.

## Figures and Tables

**Table 1:** Comparison of advantage and disadvantage of ERT, HSCT and new candidate therapies for MPS IVA.

	Advantage	Disadvantage
ERT	Low risk of mortality and morbidity	High cost ($ 400,000 per year per 25 kg)
	No limitation of age	Weekly infusion
	No specialized medical facility required	Short half-life time of the enzyme (40 min-human, 2 min-mouse)
HSCT	Lower cost than ERT (approximately $ 100,000)	Risk of mortality and morbidity
	One-time permanent treatment	Limitation of age
	Continuous activity of enzyme	Specialized medical facility required
	More effect in bone pathology than ERT	GVHD
		Availability of a donor
	Advantage	Problems to overcome
SDET	Enzyme is active in neutral pH (May work in circulation and ECM)	Immunogenicity to the enzyme
	No age limitation	Optimal dose and treatment frequency
Gene therapy	One-time permanent treatment	Vector selection needs to be determined
	Continuous activity of enzyme	(optimal promoter, AAV serotype, dose etc)
	Does not require donor	Readministration is Not available
	No age limitation	Unknown duration of enzyme expression
Nanomedicine	Protection of enzyme degradation	Limitation on components to make nanoparticles
	Greater permeability through biological membranes	Optimal dose and treatment frequency
	Better efficacy to act on lysosomes	Unknown effects still in animal model
	No age limitation	
Pharmacological chaperone therapy	Wide distribution in tissues	Off-target effect
	Oral administration	Optimal dose and treatment frequency
	No immunogenecity	Unknown effects still in animal model

Abbreviation: ERT: enzyme replacement therapy, HSCT: hematopoietic stem cell transplantation, GVHD: graft versus host disease, SDET: substrate degradation enzyme thera.

## Abbreviations

AAV	Adeno-associated virus
ADL	Activity of daily living
BBB	Blood-brain barrier
BMD	Bone mineral density
BMT	Bone marrow transplantation
C6S	Chondroitin-6-sulfate
CBA	Chicken *β*-actin
CSF	Cerebrospinal fluid
CT	Computed tomography
D8	Aspartic acid octapeptide
DBS	Dried blood spot
DMB	Dimethylmethylene blue
ECM	Extracellular matrix
ELISA	Enzyme-linked immunosorbent assay
EMA	European Medicines Agency
ERT	Enzyme replacement therapy
FDA	Food and Drug Administration
FEV1	Forced expiratory volume in 1s
FVC	Forced vital capacity
GAG	Glycosaminoglycans
GALNS	N-acetylgalactosamine 6-sulfate sulfatase
GVHD	Graft versus host disease
HS	Heparan sulfate
HSCT	Hematopoietic stem cell transplantation
KS	Keratan sulfate
LC-MS/MS	Liquid chromatography tandem mass spectrometry
MPS	Mucopolysaccharidosis
MRI	Magnetic resonance imaging
NLC	Nanostructured lipid carriers
SDET	Substrate degradation enzyme therapy
